# VAMP8 phosphorylation regulates lysosome dynamics during autophagy

**DOI:** 10.1080/27694127.2022.2031378

**Published:** 2022-03-31

**Authors:** Lei Wang, Jiajie Diao

**Affiliations:** Department of Cancer Biology, University of Cincinnati College of Medicine, Cincinnati, OH 45267, USA

**Keywords:** Autophagy, fusion, lysosomes, phosphorylation, VAMP8

## Abstract

In the final critical step for autophagic degradation, lysosomes fuse with autophagosomes to form autolysosomes. Although recent research has suggested that soluble N-ethylmaleimide-sensitive factor attachment protein receptor (SNARE) proteins are important for lysosome-autophagosome fusion, neither the architecture of the prefusion state nor the regulatory mechanisms have been identified. In our study, using structured illumination microscopy, we observed that lysosomes formed clusters around individual autophagosomes, thereby setting the stage for membrane fusion. Moreover, VAMP8 (vesicle-associated membrane protein 8) assists in forming the prefusion state of these clusters. We also found that VAMP8 phosphorylation reduces spontaneous lysosome-autophagosome fusion, whereas its dephosphorylation promotes fusion events between lysosomes and autophagosomes in both normal and autophagy-induced conditions. Our data thus suggest a key role of VAMP8 phosphorylation in the regulation of lysosome-autophagosome fusion.

Structured illumination microscopy (SIM) is a type of super-resolution microscopy that bypasses the diffraction limit in light microscopy and enables the visualization of otherwise unobservable details in biological systems. By employing SIM, we observed a dynamic process in which single dispersed lysosomes cluster when autophagy is induced by rapamycin, nutrient starvation, the ATP synthesis inhibitor oligomycin, or mitochondrial depolarization with carbonyl cyanide *m*-chlorophenylhydrazone (CCCP) [1]. In particular, we observed that multiple lysosomes bind to one autophagosome to form a cluster, thereby revealing that lysosome clusters are associated with autophagosomes. Our data suggest that lysosomes form clusters once autophagy is induced, and we confirmed these results with negative-stain electron microscopy. To analyze the lysosome-autophagy association in greater detail, we developed an algorithm to process SIM images of both organelles. Therein, lysosomes and autophagosomes were defined as being associated when the distance between them was less than 200 nm, and as being fused when they overlapped. Using those definitions, we quantified lysosome-autophagosome interactions before and after treatment with CCCP. After the treatment, we observed more events of lysosome-autophagosome contact and fusion, which implies a role of autophagosomes in guiding lysosome clustering when autophagy is induced.

To verify the association of lysosome clustering with autophagosomes, we knocked out endogenous *Atg13* (autophagy related 13) or *Rb1cc1/Fip200* (RB1-inducible coiled-coil 1) to block the formation of autophagosomes. Lysosome clustering is reduced dramatically in *atg13^−/−^* and *rb1cc1^−/−^* knockout cells compared to the wild type when autophagy is induced with CCCP, thereby suggesting that lysosome clustering depends indeed on autophagosomes.

Because VAMP8 is involved in lysosome-autophagosome fusion, we speculated that it may play a role in guiding lysosomes towards autophagosomes. To test this hypothesis, we knocked down endogenous VAMP8. The percentage of lysosome-autophagosome association and fusion decrease in VAMP8 knockdown cells when different autophagy inducers are used. The lessening in lysosome clustering, however, does not affect the number of lysosomes. We also used negative-stain electron microscopy to verify the reduction in lysosome clustering caused by VAMP8 knockdown. The formation of autolysosomes also decreases upon VAMP8 knockdown. These data suggest that lysosome clusters are important for the prefusion state between lysosomal and autophagosomal membranes.

To investigate the mechanisms behind the fusion of lysosome clusters with autophagosomes, we additionally examined the role of VAMP8 phosphorylation. Three phosphorylation sites of VAMP8, i.e., T48, T54, and S55, were mutated to glutamic acid (VAMP8[3E]) to mimic its phosphorylation. An in vitro lipid-mixing assay revealed that the VAMP8[3E] triple mutant reduces the heterotypic fusion of proteoliposomes reconstituted with VAMP8 and STX17 (syntaxin 17) plus SNAP29 (synaptosomal-associated protein 29), thereby implying the inhibitive role of VAMP8 phosphorylation in fusion events. To confirm this notion at the cellular level, a triple mutant VAMP8 (VAMP8[3A]) was generated to prevent this protein from being phosphorylated in vivo. Consistently, the number of lysosome-autophagosome fusion events increased in cells expressing VAMP8[3A]. Figure 1 depicts the putative role of VAMP8 phosphorylation in those events. Because autophagy is known to determine cell fate, we also examined the role of VAMP8 phosphorylation in resistance to temozolomide, a drug that is used to treat brain cancer for chemotherapy. The data show that VAMP8 silencing and the phosphorylation-defective mutant of VAMP8 both reduce resistance to temozolomide. Thus it implies that VAMP8 phosphorylation plays an important role in temozolomide resistance by influencing autophagic flux.

**Figure 1.**
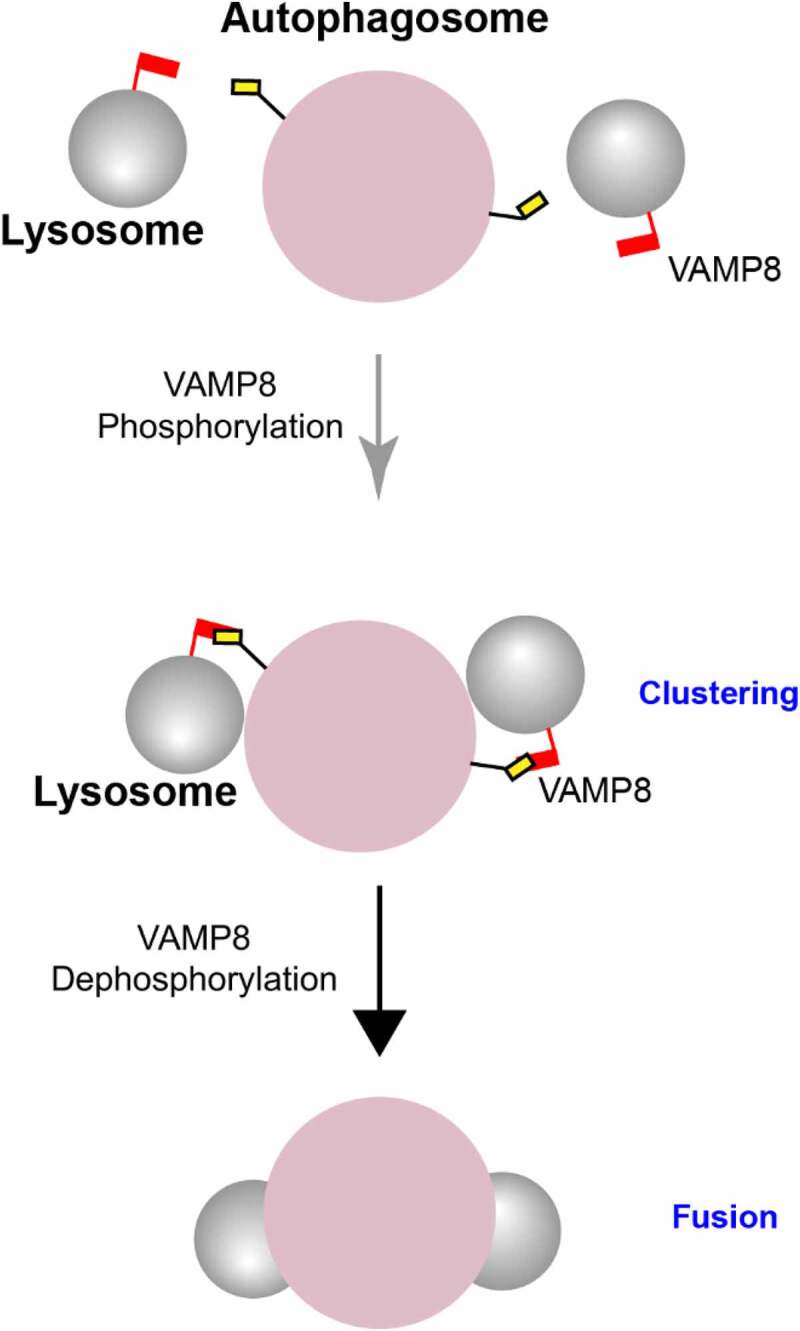
Schematic of VAMP8-regulated association of lysosomes with autophagosomes. The phosphorylation of VAMP8 inhibits lysosome-autophagosome fusion, whereas its dephosphorylation promotes lysosome-autophagosome fusion.

Taken together, we showed that lysosomes form clusters around autophagosomes for the initiation of membrane fusion, and the fusion of lysosomes with autophagosomes is regulated by the phosphorylation of VAMP8.
